# Effect of Baicalin-Aluminum Complexes on Fecal Microbiome in Piglets

**DOI:** 10.3390/ijms20102390

**Published:** 2019-05-14

**Authors:** Shulin Fu, Feng Zhuang, Ling Guo, Yinsheng Qiu, Jianglin Xiong, Chun Ye, Yu Liu, Zhongyuan Wu, Yongqing Hou, Chien-An Andy Hu

**Affiliations:** 1 Hubei Key Laboratory of Animal Nutrition and Feed Science, Wuhan Polytechnic University, Wuhan 430023, China; fushulin2016@126.com (S.F.); zhuang572541169@163.com (F.Z.); guoling1101@163.com (L.G.); xiongjianglin@126.com (J.X.); yechun0226@163.com (C.Y.); lyywfy@foxmail.com (Y.L.); zhongywu@163.com (Z.W.); houyq@aliyun.com (Y.H.); AHU@salud.unm.edu (C.-A.A.H.); 2 Hubei Collaborative Innovation Center for Animal Nutrition and Feed Safety, Wuhan 430023, China; 3 Biochemistry and Molecular Biology, University of New Mexico School of Medicine, Albuquerque, NM 87131, USA

**Keywords:** baicalin–aluminum complexes, gut microbiomes, diarrhea

## Abstract

The gut microbiome has important effects on gastrointestinal diseases. Diarrhea attenuation functions of baicalin (BA) is not clear. Baicalin–aluminum complexes (BBA) were synthesized from BA, but the BBA’s efficacy on the diarrhea of piglets and the gut microbiomes have not been explored and the mechanism remains unclear. This study has explored whether BBA could modulate the composition of the gut microbiomes of piglets during diarrhea. The results showed that the diarrhea rate reduced significantly after treatment with BBA. BBA altered the overall structure of the gut microbiomes. In addition, the Gene Ontology (GO) enrichment analysis indicated that the functional differentially expressed genes, which were involved in the top 30 GO enrichments, were associated with hydrogenase (acceptor) activity, nicotinamide-nucleotide adenylyltransferase activity, and isocitrate lyase activity, belong to the molecular function. Kyoto Encyclopedia of Genes and Genomes (KEGG) enrichment analysis showed that flagellar assembly, bacterial chemotaxis, lipopolysaccharide biosynthesis, ATP-binding cassette transporters (ABC) transporters, biosynthesis of amino acids, and phosphotransferase system (PTS) were the most enriched during BBA treatment process. Taken together, our results first demonstrated that BBA treatment could modulate the gut microbiomes composition of piglets with diarrhea, which may provide new potential insights on the mechanisms of gut microbiomes associated underlying the antimicrobial efficacy of BBA.

## 1. Introduction

Post-weaning diarrhea (PWD) is thought to be one of the most important diseases for the modern pig industry worldwide because it causes serious financial losses [1]. There are many factors related to diarrhea of piglets. Enterotoxigenic *Escherichia coli* (*E. coli*) (ETEC) is generally considered to be an important factor associated with PWD of piglets [2]. In addition, previous reports showed that stress factors during weaning period might negatively regulate the response to immune system which could result in alteration of intestinal flora and dysfunction [3,4]. Antibiotics are commonly utilized for controlling PWD of piglets [5]. However, there is a concern that long-term utilization of antibiotics may lead to drug resistance [6] and risk of residue in the human body through food transmission [7]. Therefore, currently developing a new Chinese herbal medicine has become an important approach to solve the diarrhea in piglets.

Baicalin–aluminum complexes (BBA) are synthesized from baicalin. Baicalin is an important effective plant-derived compound of the traditional Chinese medicine, which is extracted from *Scutellaria baicalensis* Georgi (Huang Qin) [8]. Baicalin has been shown exhibiting important biological functions. Baicalin could provide protection against *Salmonella typhimurium* infection, reduce the bacterial virulence, and modulate host response [9]. Baicalin significantly attenuated the production of HMGB1 in peripheral blood monocytes induced by *Haemophilus parasuis* during inflammation process [10]. Baicalin triggered the activation of autophagy in the macrophages stimulated by *Mycobacterium tuberculosis* infection via PI3K/Akt/mTOR pathway, thus producing anti-inflammatory function [11]. It has been documented that baicalin could inhibit the lethality of Shiga-like toxin 2 [12] and provide significant protection to mice from the challenge of Shiga-like toxin-producing *Escherichia coli* (STEC) O157:H7 [13]. In addition, baicalin has shown an effect on inhibiting growth and reducing antimicrobial resistance of *Escherichia coli* [14]. Alum contains aluminum sulfate and has been shown to possess good antibacterial activity [15]. It has been documented that a commercial product, Huangqinsulv capsule, of which the main active ingredient is baicalin-aluminium complex, was synthesized by using alum and the extracts of *Scutellaria baicalensis* Georgi [16]. Huangqinsulv capsule was utilized clinically to treat human diarrhea for more than 30 years. However, whether BBA could inhibit bacterial virulence-related factors and modulate the composition of the gut microbiome of piglets during diarrhea has not been deeply studied.

Previous research has reported that dynamic gut microbiomes have key effects on maintaining balance and host’s health, may be through crosstalk between host immunity and microorganism [17,18]. If the dynamic balance of the interaction between host and microbe is broken, intestinal inflammation diseases such as PWD of piglets might occur [19]. It has been recorded that a disorder of gastrointestinal flora balance could cause inflammatory bowel disease [20]. Therefore, regulation of the gut microbiomes may serve as a target to control some diseases, such as nonalcoholic fatty liver disease (NAFLD) [21]. Metagenomic analysis provides a favorable platform for understanding changes of gut flora during disease processes. The technology could exactly reveal more active microbial communities [22].

In this study, thirty piglets with PWD were chosen to explore the efficacy of BBA on the treatment of diarrhea and the structural changes of the gut microbiomes in response to the treatment with BBA. The results demonstrated that the rate of diarrhea among piglets reduced significantly after treatment with BBA, and BBA could modulate the composition of gut microbiomes of piglets by metagenomic analysis, which may provide new potential insights on the mechanisms of gut microbiomes associated underlying the anti-microbiological efficacy of BBA.

## 2. Results

### 2.1. BBA Significantly Reduced the Diarrhea Rates of Piglets

**Figure 1:**
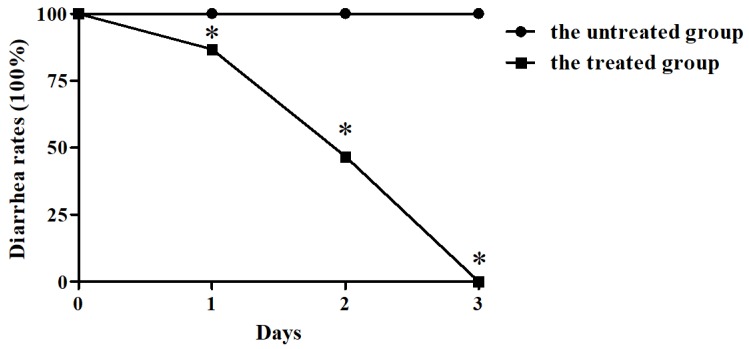
Baicalin–aluminum complexes (BBA) significantly inhibited the diarrhea rates of piglets. * indicates significance at *p* < 0.05.

During clinical observation, on the day with the piglets administrated with BBA, the diarrhea rates of the piglets significantly reduced compared with the untreated group (Figure 1) (*p* < 0.05). Furthermore, on the third day, no clinical symptoms of piglets with BBA treatment were observed, but the untreated group still displayed diarrhea (Figure 1).

### 2.2. Overall Structural Modulation of Gut Microbiome Following Treatment with BBA

In this study, the metagenomic sequencing analysis was used to detect the structural changes in the gut microbiomes in the two groups before and after treatment with BBA or not. The results showed that high-throughput metagenomic sequencing produced 562,699,940 clean reads from 6 stool samples with 93,783,323 ± 11,256,477 sequences per sample (Table 1, Supplemental Table S1).

**Figure 2:**
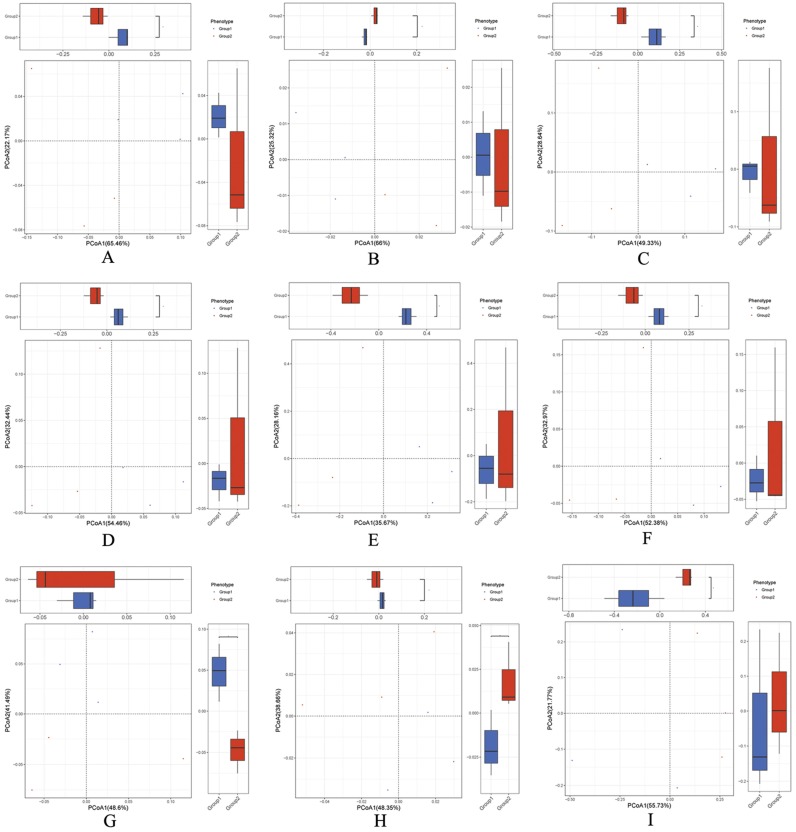
Principal coordinates analysis (PCoA) of effect of baicalin–aluminum complexes (BBA) on the gut microbiomes. PCoA of the ARDB changes (**A**), category changes (**B**), Non-supervised Orthologous Groups (eggNOG) changes (**C**), enzyme changes (**D**), gene changes (**E**), KO changes (**F**), module changes (**G**), pathway changes (**H**), and species of taxonomy changes (**I**) of the gut microbiomes in different groups. Group 1: The three piglets with diarrhea not treated with BBA (sample erqian, sample sanqian, sample siqian); Group 2: The same three piglets with diarrhea treated with BBA (sample erhou, sample sanhou, sample sihou).

Unweighted UniFrac PCoA was utilized to detect the composition of microbiomes from different groups in view of the evolutionary distance [23]. PCoA analysis showed that BBA treatment group and control group displayed changes, and a distinct cluster of the piglets’ gut microbiome composition from BBA treatment group was existed (Figure 2). Additionally, BBA treatment group were clearly separated from the two groups in this study (Figure 2). Furthermore, BBA treatment group has shown an effect on the overall gut microbiomes compositions of piglets with diarrhea (Figure 2).

**Figure 3:**
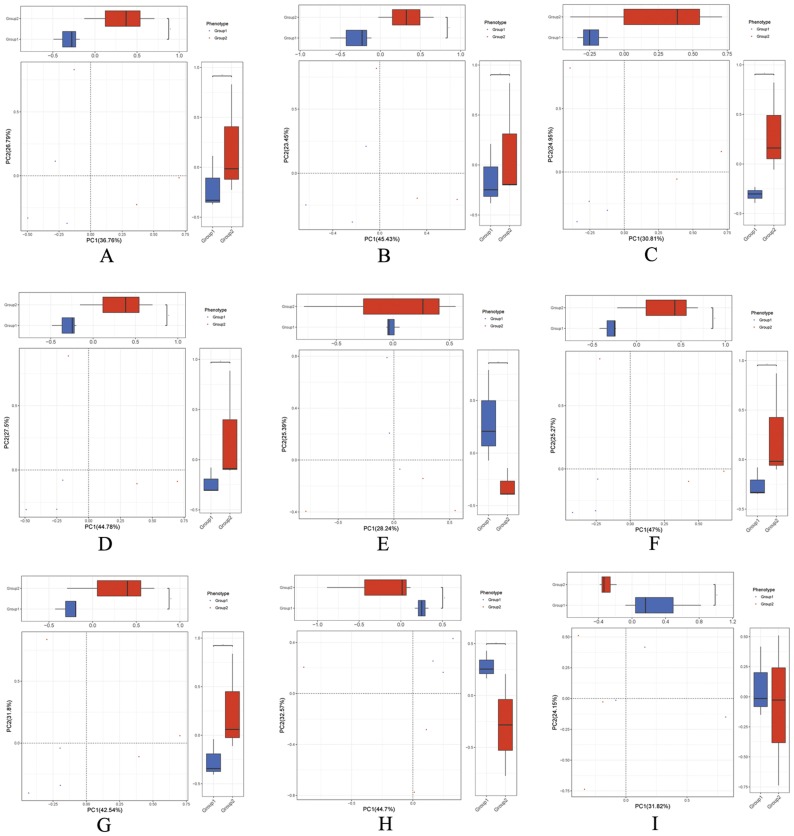
Principal components analysis (PCA) of BBA effect on the gut microbiomes. PCA of the ARDB (**A**), category (**B**), eggNOG (**C**), enzyme (**D**), gene (**E**), KO (**F**), module (**G**), pathway (**H**), and species of taxonomy (**I**) of the gut microbiomes in different groups. Group 1: The three piglets with diarrhea not treated with BBA (sample erqian, sample sanqian, sample siqian); Group 2: The same three piglets with diarrhea treated with BBA (sample erhou, sample sanhou, sample sihou).

Principal components analysis (PCA) analysis method was utilized to explore the relationship between the numerous samples and multivariate data [24]. The results demonstrated that most of the samples from BBA treatment group and control group emerged a mixed distribution with not falling into a single cluster (Figure 3). Furthermore, we found that the Non-supervised Orthologous Groups (eggNOG), module, and species of taxonomy changes of samples exhibited some disperse, but they were fully distinguished from each other (Figure 3C,G,I).

### 2.3. The Effect of BBA on Modulation of Important Phylotypes of Gut Microbiome

**Figure 4:**
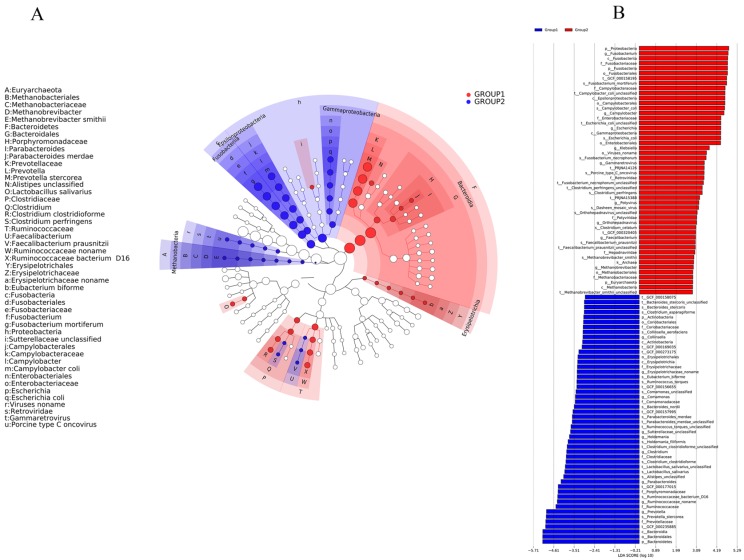
Species community structure diagram of gut microbiomes of the piglets by linear discriminant analysis effect size (LEfSe). The nodes size displayed the abundance of the species. Group 1: The three piglets with diarrhea not treated with BBA (sample erqian, sample sanqian, sample siqian); Group 2: The same three piglets with diarrhea treated with BBA (sample erhou, sample sanhou, sample sihou).

**Figure 5:**
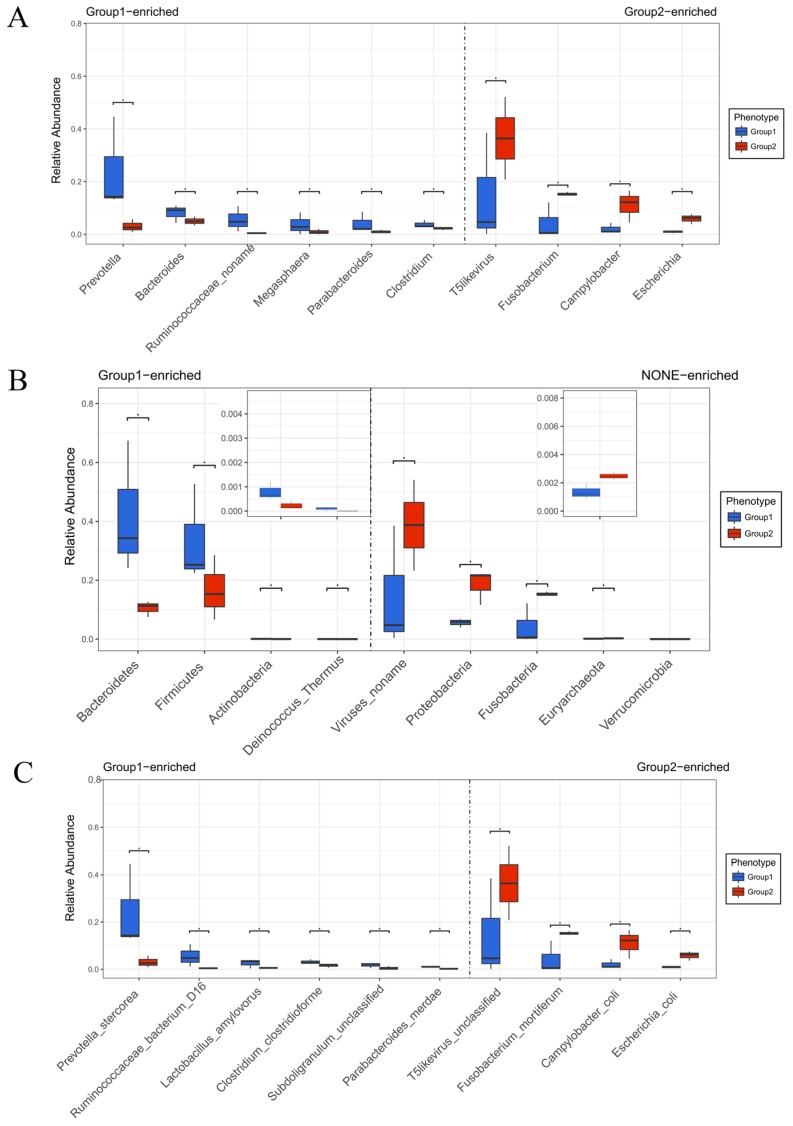
The genus level (**A**), phylum level (**B**), and species level (**C**) detection based on taxonomy analysis in the piglets. The results of genus level, phylum level, and species level were showed on the y-axis in each of the panels. Group 1: The three piglets with diarrhea not treated with BBA (sample erqian, sample sanqian, sample siqian); Group 2: The same three piglets with diarrhea treated with BBA (sample erhou, sample sanhou, sample sihou).

From the species community structure diagram, we found that *Bacteroidetes*, *Clostridiaceae*, and *Ruminococcaceae* were the main microbiomes which exhibited the most important components of the piglets’ gut microbiomes with diarrhea (Figure 4). After the piglets’ treatment with BBA, the microbiomes were changed and *Proteobacteria*, *Euryarchaeota*, and *Fusobacteria* were the most important components of gut microbiomes of piglets, which suggested that BBA could modulate the components of gut microbiomes (Figure 4). The genus level, phylum level, and species level were shown in Figure 5, Supplemental Figure S1. The phylum level of taxonomy analysis demonstrated that *Viruses noname*, *Proteobacteria*, *Euryarchaeota*, and *Fusobacteria* were increased, while *Firmicutes*, *Actinobacteria,* and *Bacteroidetes* were decreased in the piglets treated with BBA compared with before BBA administration (Figure 5B).

**Figure 6:**
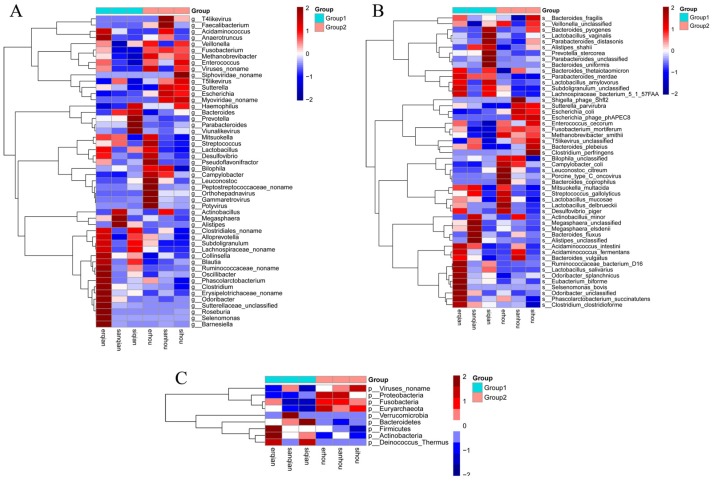
The heatmap of taxonomy analysis at the genus level (**A**), phylum level (**B**), and species level (**C**). Group 1: The three piglets with diarrhea not treated with BBA) (sample erqian, sample sanqian, sample siqian); Group 2: The same three piglets with diarrhea treated with BBA (sample erhou, sample sanhou, sample sihou).

The heatmap of taxonomy comparison analysis at the genus, phylum, and species level were also displayed. The results demonstrated that, at the genus level, *Fusobacterium*, *Lactobacillus*, *Prevotella*, and *Escherichia* were predominant in each group (Figure 6A). After the piglets treated with BBA, the *Prevotella*, *Lactobacillus,* and *Bacteroides* were significantly reduced (Figure 6A). However, *Fusobacterium* and *Campylobacter* were increased (Figure 6A). In addition, *mortiferum* from *Fusobacterium coli* from *Campylobacter* and *Escherichia* significantly increased at the species level, while *stercorea* from *Prevotella delbrueckii* from *Lactobacillus* decreased compared with piglets before BBA administration (Figure 6C).

### 2.4. The Effect of BBA on Modulation of Genes Expression of Gut Microbiome

To better understand the microbe response to BBA treatment, the enrichment analysis using Database for Annotation, Visualization, and Integrated Discovery (DAVID) was performed. The GO enrichment analysis demonstrated that the functional differentially expressed genes which were involved in the top 30 GO enrichments were associated with hydrogenase (acceptor) activity, nicotinamide-nucleotide adenylyltransferase activity, and isocitrate lyase activity, belonged to the molecular function (Supplemental Figure S2A). Choline metabolic process, cell adhesion involved in single-species biofilm formation, and arabinose transport belonged to the biological process (Supplemental Figure S2A). Viral assembly intermediate belonged to the cellular component (Supplemental Figure S2A). Further, the top 30 pathways screened as enrichment in the microbiomes of the piglets treated with BBA by KEGG were displayed in Supplemental Figure S2B. Flagellar assembly, bacterial chemotaxis, lipopolysaccharide biosynthesis, ABC transporters, biosynthesis of amino acids, and phosphotransferase system (PTS), were the most enriched during BBA treatment process, which suggested the key importance of the signaling pathways upon gut microbiomes modulation.

## 3. Discussion

In this study, BBA significantly reduced diarrhea compared with the control group; therefore, it could be used in effective treatment of piglets with diarrhea. Previous research has reported that BA could attenuate the diarrhea in rats and pathological lesions of intestine induced by food allergy [25]. The active compound, baicalin, displayed effectiveness in the treatment of bovine viral diarrhea (BVD) which may inhibit inflammation and enhance immune responses in the host during virus infection process [26]. In addition, baicalin could improve the infections diarrhea of piglets which change the metabolism of components in the diarrheal intestinal flora [27]. BBA was synthesized from BA, but the BBA’s efficacy on the gut microbiomes has not been explored. In this study, BBA displayed significant efficacy on inhibiting diarrhea of piglets, and we first demonstrated that BBA modulated the overall gut microbiomes compositions of piglets with diarrhea, which might be served as a promising candidate for use in the diarrhea treatment.

Although BBA exhibited excellent effect on diarrhea among piglets, the anti-diarrhea mechanism of BBA remains unclear. Few researches showed that occurrence of diarrhea may be associated with the imbalance of intestinal flora [28,29]. On the third day, no clinical symptoms in piglets with BBA treatment were observed, so we collected the stool samples for metagenomic sequencing on the third day. We speculated that BBA could modify the gut microbiomes composition resulting in attenuation of diarrhea. As shown in this study, BBA significantly changed the gut microbiomes structure and modulated the composition of gut microbiomes at taxonomic levels. The phylum *Bacteroidetes* and *Firmicutes* were decreased following treatment with BBA. It has been documented that vanillin could reduce the abundance of *Firmicutes* phylum related with the obesity induced by high-fat-diet (HFD) [30], and rutin also could inhibit the HFD-stimulated increase of *Bacteroidetes* [31], which was consistent with our results. In addition, we found that BBA could also affect the changes at the species level. Previous research has shown that administration of *Lactobacillus amylovorus* could lead to free ammonia removal in intestinal lumen, which contributes to the blood ammonia levels attenuation in the host [32]. *Lactobacillus amylovorus* has good antiviral property against enterovirus infection [33]. *Lactobacillus amylovorus* could inhibit the *E. coli* adherence to porcine intestinal epithelial cells (IPEC) and stimulate the production of anti-inflammatory cytokines in human dendritic cells (DCs) [34]. It has been documented that oral supplementation with live *Lactobacillus salivarius* could induce growth performance and gut health, attenuate diarrhea incidence, and promote intestinal morphology of sucking pig [35]. *Lactobacillus salivarius* inhibited the biofilm formation and downregulated the levels of genes expression involved in exopolysaccharide secretion, acid tolerance, and quorum sensing of *Streptococcus mutans* [36]. In this study, the abundance of *Lactobacillus salivarius* and *Lactobacillus amylovorus* were significantly decreased following BBA treatment, which might be used and converted by the host. Based on the previous research and our findings, we speculated that BBA could be utilized in selective modulations of gut microbe phenotype involved in the beneficial efficacy in alleviating diarrhea to obtain dynamic adjustment or balance in flora, ultimately affecting the interaction between microbe and host. Further evidences need to be deeply studied the interaction between host and microbiomes.

Interestingly, after treatment with BBA, the *T5-like viruses* (*T5-like phages*) were significantly increased compared with before BBA administration. It has been reported that *bacteriophage* is related to gut microbiomes and have important effects on community structure and function [37]. *Bacteriophage* could change the functions of the microbiomes community by selective elimination of species of the gut microbiomes [38], leading to the modification of the host immune system [39]. In addition, use of *bacteriophage* transplants could reestablish the health of human gut, which demonstrated that *bacteriophage* might be served as disease treatment [40,41]. In this study, we first reported that *T5-like viruses* existed in the piglets’ gut. Although the role of *bacteriophages* in the piglets’ gut is poorly understood, we inferred that the increased active *bacteriophages* may have important effects on maintaining the functions and structures of the gut microbiomes balance.

In this study, BBA treatment of piglets with diarrhea resulted in functional change of gut microbiomes. Among the signaling pathways, ATP-binding cassette transporters (ABC transporters) were more dynamic in gut microbiomes from piglets treated with BBA, and 202 of 226 genes were significantly downregulated in this signaling. Studies have reported that ABC transporters belong to the members of transport system super family which exist in all extant phyla from prokaryotes to eukaryotes. ABC transporters on the cell membrane could be classified as exporters [42] or importers which rely on the direction of transportation against the cytoplasm [43]. The ABC transporters have been shown to be related to the occurrence of multidrug resistance [44], human diseases such as cancer [45], and inflammatory bowel disease [46]. The abundance of ABC transporters enrichment is increased after treatment with BBA, which might be associated with advance of absorption of nutrients, vitamins, and metabolites [47]. Our study first found that ABC transporters were related to diarrhea of piglets following treatment with BBA, but the mechanism will be further studied in our next work. The unique features of phosphotransferase system (PTS) were constituted of phosphoenolpyruvate (PEP) as the phosphoryl donor, termed Enzyme I, Enzyme II, and HPr, which were essential for catalytic entities [48]. It has been documented that PTS has important functions in regulation of transport, metabolism, and genes expression [49]. PTS also participate in the stimulation of biofilm formation, swarming motility, and induction of root colonization of bacteria [50]. Through functional analysis, we indicated that PTS was possibly participated in regulating the gut microbiomes metabolism. It has been reported that bacterial virulence-related factors were involved in the flagellar assembly [51], bacterial chemotaxis [52,53], and lipopolysaccharide biosynthesis [54]. In addition, the genes in the flagellar assembly, bacterial chemotaxis, and lipopolysaccharide biosynthesis signaling pathways were most downregulated following BBA treatment. Therefore, we presumed that BBA might inhibit the expression of bacterial virulence-related factors, metabolism and transport of amino acid, thereby attenuating the diarrhea of piglets.

Taken together, our results first showed that BBA could modulate the changes of gut microbe communities and subsequently contributed to alleviating diarrhea of piglets. Our study found a new effect of BBA in easing the rate of diarrhea, which may provide a novel therapy target for controlling diarrhea of piglets.

## 4. Materials and Methods

### 4.1. Drugs

Baicalin-aluminum complexes (BBA) are synthesized by Institute of Animal Health Products, Wuhan Polytechnic University. The BBA was obtained by the methodology which was used to prepare Huangqinsulv capsules (WS3-B-1425-93). BBA was prepared by the reaction of an extraction of *Scutellaria baicalensis* Georgi and alums in water, at pH 7.0 (regulated by Na_2_CO_3_), under constant stirring for 15 min. Following filtration and washing with water and ethyl alcohol, the BBA was isolated as an orange powder and being dried in a desiccator under vacuum. When used, 13.6 g BBA per pack was dissolved using 50 mL normal saline. 

### 4.2. Collection of Stool

This study was carried out in strict accordance with the recommendations in the China Regulations for the Administration of Affairs Concerning Experimental Animals 1988 and the Hubei Regulations for the Administration of Affairs Concerning Experimental Animals 2005. This study was performed in a commercial farm. Thirty 10-day-old, weighing 4–4.3 kg, naturally farrowed early-weaned (NFEW) piglets (Duroc × Landrace × large white) which were suffering from diarrhea were chosen and used for in vitro experiments. The piglets with diarrhea were randomly divided into two groups. Five mL normal saline which contained BBA 1.36 grams was given to piglets from BBA treated group by intragastric administration for 3 days, twice a day, continuously. BBA untreated group was administered with 5 mL normal saline as the control group. The stools from three piglets (sample named erqian, sanqian, siqian) untreated with BBA were collected, and the stools from the same three piglets (sample named erhou, sanhou, sihou) treated with BBA were collected at 3 day for metagenomic sequencing. 

### 4.3. DNA Extraction

DNA purification from stool was carried out according to the protocols [55]. In brief, the stools from the piglets were collected in storage tubes and then stored in liquid nitrogen until DNA extraction. The genomic DNA was extracted from stools by using the QiagenQIAamp DNA Stool Mini Kit (Qiagen, Valencia, CA, USA) for DNA sequence analysis (DNA-Seq) in the Shanghai Biochip Corporation (Shanghai, China) according to the manufacturer’s instructions. The concentration and molecular weight of the genomic DNA were determined by utilizing the nanodrop instrument (Thermo Scientific, Waltham, MA, USA) and agarose gel electrophoresis, respectively.

### 4.4. DNA Library Construction and Sequencing

DNA library construction was carried out by using the NEBNext Ultra DNA Library Prep Kit for Illumina (NEB, Ipswich, MA, USA) according to the manufacturer’s instruction. The library quality was detected utilizing Qubit dsDNA HS Assay Kit (Invitrogen, Carlsbad, CA, USA). The paired-end metagenomic sequencing (2 × 150 base pair) was determined in the Illumina platform.

### 4.5. Metagenomic Analysis

The sequencing reads were quality performed and assembled de novo in contigs by utilizing metaSPAdes (SPAdes-3.10.1) [56]. Gene prediction with the default parameters was carried out by using MetaGeneMark (GeneMark.hmm version 3.38) [57]. Redundant genes were deleted by utilizing Blastn software with the cutoff of 90% length and 95% identity [58]. Relative abundances of the genes were performed by aligning high-quality sequencing reads to the gene catalog [59]. The putative amino acid sequences were aligned against the proteins/domains within evolutionary genealogy of genes: Non-supervised Orthologous Groups (eggNOG) (v4.5) and Kyoto Encyclopedia of Genes and Genomes (KEGG) databases (release 59.0) by utilizing diamond (e-value ≤ 1 × 10^−5^) [60]. To determine the abundances of eggNOG or KEGG orthologue groups, the abundances of proteins were added which were assigned into the same eggNOG or KEGG orthologue groups. The quality-controlled reads were carried out using MetaPhlAn2 to obtain the taxonomic profile of the microbial community [61]. 

eggNOGs were characterized by determining their enrichment analysis using the Gene Ontology (GO) classification. The biological pathways involved in eggNOGs were analyzed using Database for Annotation, Visualization, and Integrated Discovery (DAVID), version 6.7. GO and pathway enrichment analysis were determined using DAVID. UniFrac-based principal coordinates analysis (PCoA) was detected by QIIME (V1.8.0) [62]. Principal components analysis (PCA) was determined using R (3.0.2) [63]. Linear discriminant analysis effect size (LEfSe) software was used to identify the differences of representative species, Kos, and eggNOGs between two or more groups of the samples under different conditions [64].

### 4.6. Statistical Analysis

The experimental data were presented as mean ± SD. The difference between two groups was analyzed using the two-tailed Student *t*-test. *p* values of <0.05 were considered significant. * *p* < 0.05.

## 5. Conclusions

Our data showed that BBA could significantly reduce the diarrhea rates among piglets, and modulate the composition, phylotypes, and genes expression of gut microbiomes. Our results may provide new potential anti-microbiological mechanisms of BBA and a novel therapy target to control diarrhea of piglets.

## Figures and Tables

**Table 1:** Statistical summary analysis of gut microbiome.

Samples	Raw Reads	Clean Reads	Clean Ratio (%)	Mapped Ratio (%)
Treated with BAA	89,537,684 ± 5,405,796.2	87,247,590.3 ± 5,144,406.2	94.9 ± 0.2	99.1 ± 0.1
Untreated	103,588,655.3 ± 13,288,842.4	100,319,056.3 ± 12,734,141.2	92.6 ± 0.2	99.0 ± 0.1

## Supplementary Materials

Supplementary materials can be found at https://www.mdpi.com/1422-0067/20/10/2390/s1.
