# Supplementary material for: Effect of Baicalin-Aluminum Complexes on Fecal Microbiome in Piglets
Source: Int J Mol Sci. 2019 May 14;20(10):2390. doi: 10.3390/ijms20102390 (PMC6566245; doi:10.3390/ijms20102390)
Supplement: Supplementary file 1 [file ijms-20-02390-s001.pdf]

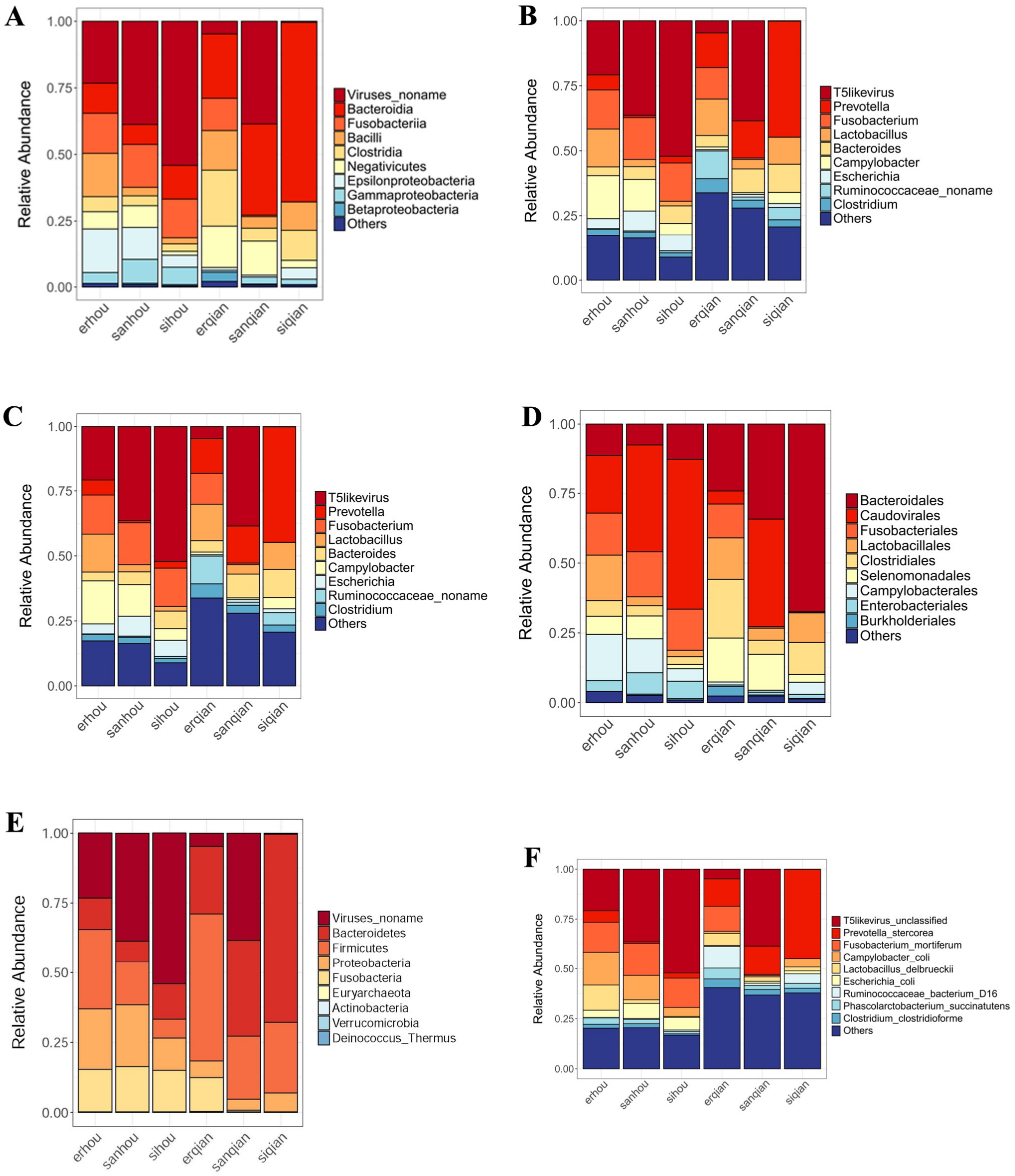

Supplemental figure S1: The class level (A), family level (B), genus level (C), order level (D), phylum level (E) and species level (F) detection in stackedbar based on taxonomy analysis in the piglets. Group 1: the three piglets with diarrhea not treated with BBA (sample erqian, sample sanqian, sample siqian); Group 2: the same three piglets with diarrhea treated with BBA (sample erhou, sample sanhou, sample sihou).

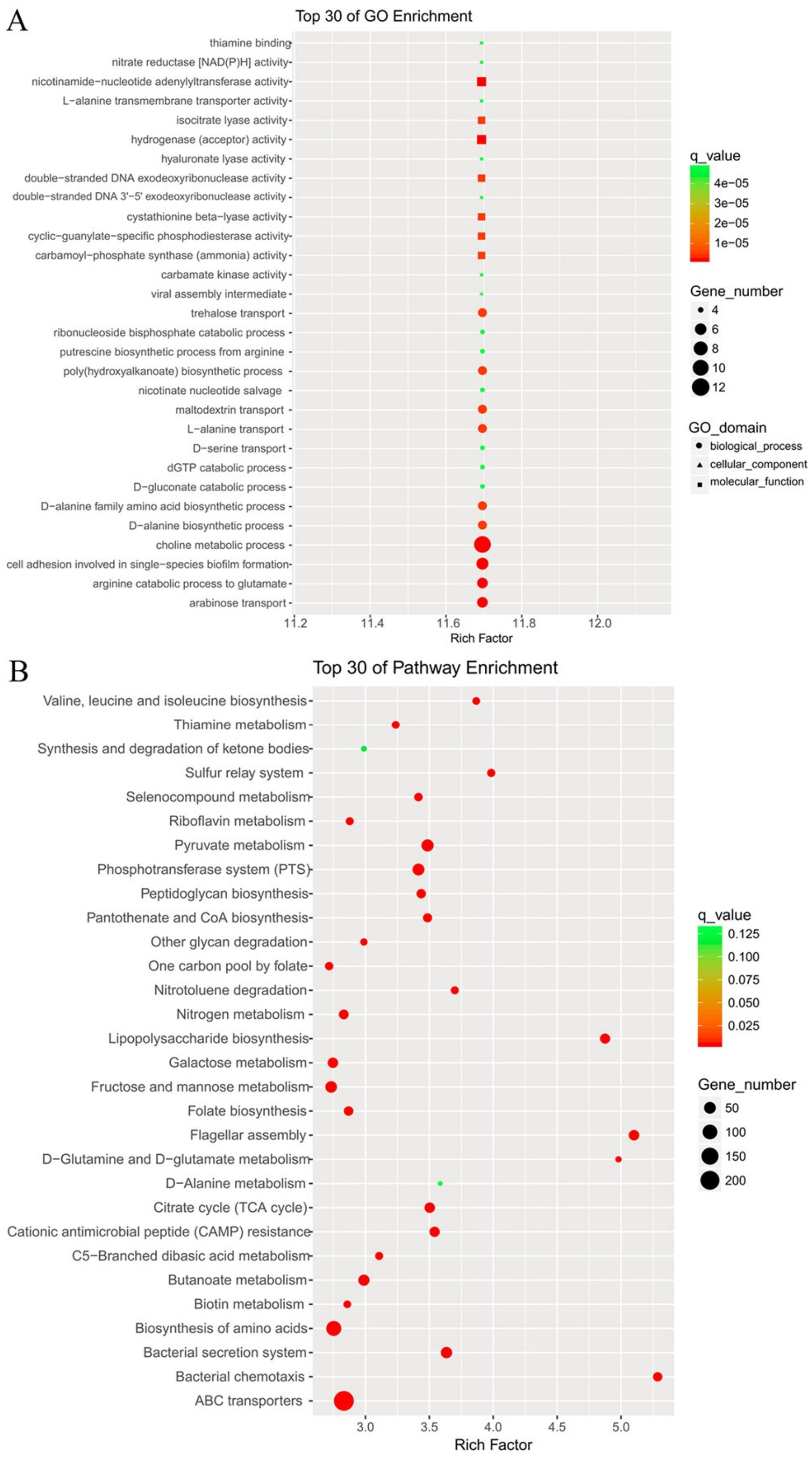

Supplemental figure S2. Analysis of the top 30 GO enrichment (A) and top 30 pathway enrichment (B) by DAVID. eggNOGs were characterized by determining their enrichment analysis using the GO classification. GO and pathway enrichment analysis were determined using DAVID.
